# Antimicrobial use in breeding kennels and antimicrobial resistance profile of *Escherichia coli* and *Staphylococcus pseudintermedius* isolated from healthy breeding bitches in Northern Italy

**DOI:** 10.3389/fvets.2025.1703350

**Published:** 2026-01-12

**Authors:** Chiara Milani, Alice Diana, Michela Corrò, Elena Spagnolo, Angela Del Carro, Ada Rota, Alessia Bertero

**Affiliations:** 1 Department of Animal Medicine, Production and Health, University of Padova, Padova, Italy; 2 Federation of Veterinarians of Europe, Brussels, Belgium; 3 Istituto Zooprofilattico Sperimentale delle Venezie, Legnaro, Padova, Italy; 4 Department of Veterinary Sciences, University of Turin, Grugliasco, Torino, Italy

**Keywords:** antimicrobial pressure, antimicrobial resistance (AMR) profile, breeding, canine, *Escherichia coli*, *Staphylococcus pseudintermedius*

## Abstract

Antimicrobials can be overused in dog breeding facilities, leading to increased resistance among commensal bacteria. The aim of this study was to investigate antimicrobial use intensity in a professional canine reproduction setting by assessing the resistance profiles of *Escherichia coli* and *Staphylococcus pseudintermedius* isolated from healthy breeding bitches, as well as through a questionnaire for breeders. Five bitches from each of 15 kennels in Northern Italy were sampled from the perivulvar skin and rectum, and the susceptibility of the isolated bacteria to a panel of different antimicrobials was determined (Minimum Inhibitory Concentration). Kennels were classified according to their reported intensity of antimicrobial use, and the association between antimicrobial use and resistance in *S. pseudintermedius* and *E. coli* was evaluated using Fisher’s exact test (*p* < 0.05). *E. coli* exhibited the highest resistance to ampicillin (around 50%), moderate resistance to amoxicillin-clavulanic acid (~24%) and cefalexin (~47%), while resistance to the remaining agents was low (generally ≤10–15%); the prevalence of Extended Spectrum Beta-Lactamase (ESBL)-producing *E. coli* was 7.76%. More than 82% of *S. pseudintermedius* strains were resistant to penicillin and ampicillin; *mec*A-positive methicillin-resistant *S. pseudintermedius* accounted for 17.65%. Multi-drug resistant (MDR, i.e., acquired non-susceptibility to at least one agent in three or more antimicrobial categories) *E. coli* and *S. pseudintermedius* were 22.53 and 41.18%, respectively. MDR *E. coli* were significantly more frequent in kennels reporting ‘high antimicrobial use’ (*p* = 0.0160). The generally high levels of acquired resistance suggest extensive antimicrobial use, especially beta-lactams. Breeder responses were partly inconsistent, indicating that complementary strategies should be adopted to monitor antimicrobial use in dog breeding facilities.

## 1 Introduction

Antimicrobial resistance (AMR) is recognized as one of the most significant global health threats, accounting for an estimated 1.27 million deaths directly attributable to bacterial AMR and contributing to an additional 4.94 million deaths worldwide (1). Given its profound implications for public health and well-being, AMR has been identified as one of the major concerns by the World Health Organization (2).

The past use of antimicrobials as growth promoters in livestock is a recognized cause of the selection and fixation of resistance genes in bacteria, in addition to the therapeutic use of antimicrobials in animal agriculture that contributes to the AMR problem onset (3). A global ‘One Health’ approach is necessary to address AMR because human, animal and environmental health are strictly interconnected (3). As a major One Health challenge, AMR requires surveillance and interventions across human, animal, and environmental domains (4, 5).

Specific considerations are warranted for dog breeding kennel facilities, as housing conditions influence disease exposure, and the health management of kennel dogs differs substantially from that of privately owned dogs. Moreover, in breeding facilities, antimicrobials may be overused or misused in attempts to improve fertility and reduce neonatal mortality (6). This practice represents a concern that requires strict surveillance and antimicrobial stewardship to ensure prudent and responsible use.

The widespread use of broad-spectrum antimicrobials in companion animals constitutes a major driver of AMR (7). The selective pressure exerted on commensal microorganisms promotes resistant strains that may subsequently transfer resistance genes to pathogenic bacteria (8).

*Escherichia coli* (*E. coli*) is a widely distributed commensal of the canine intestine, while *Staphylococcus pseudintermedius* commonly colonizes dog skin and mucosae. The 100% prevalence of *E. coli* in fecal samples from healthy dogs (9) and the approximately 54% prevalence of *S. pseudintermedius* on the perineal skin of healthy dogs (10) make these two species useful indicators of antimicrobial selective pressure. When *E. coli* or coagulase-positive staphylococci acquire resistance to at least one agent in three or more antimicrobial classes, they are defined as Multi-Drug Resistant (MDR) bacteria (11).

Under selective pressure from beta-lactam antimicrobials, *E. coli* had developed resistance through beta-lactamase enzymes. The evolution of these enzymes has paralleled the introduction of first- and second-generation cephalosporins followed by oxyimino-cephalosporins (12). Extended-Spectrum Beta-Lactamase (ESBL)-producing *E. coli* are now endemic among Enterobacterales and exhibit resistance to most beta-lactam antimicrobials, including extended-spectrum cephalosporins and monobactams (12).

Since 2006, methicillin resistant *S. pseudintermedius* clones have emerged as significant pathogens in small animal medicine, and have become endemic worldwide (13). Methicillin resistance in *S. pseudintermedius*, similarly to *S. aureus*, is mediated by the *mec*A gene, which encodes an altered penicillin-binding protein (PBP). As beta-lactam antimicrobials cannot effectively bind to this modified PBP, they are unable to inhibit bacterial cell wall synthesis (14).

The aim of this study was: (a) to investigate the resistance profiles of *Escherichia coli* and *Staphylococcus pseudintermedius* isolated from healthy breeding bitches living in small- to medium-sized breeding facilities as indicators of antimicrobial pressure; (b) to investigate the antimicrobial use through a dedicated questionnaire for breeders; and (c) to correlate the breeders’ responses with bacteriological findings.

## 2 Materials and methods

### 2.1 Animals and sampling

The breeding bitches included in the study belonged to 15 breeding kennels located in two regions of Northern Italy (Piedmont and Veneto). The breeding facilities were selected according to the following predefined criteria: between 5 and 20 breeding bitches belonging to each breeding facility, at least three litters born per year, and no more than two different breeds in each facility.

Sample collection was carried out from July 2023 to November 2023 and was approved by the Ethical Committee of the Department of Veterinary Sciences of the University of Turin, Italy (Approval n° 0003085, 10/10/2023). All breeders provided written informed consent, and procedures were conducted in accordance with EU Directive 86/609/CEE and Italian Ministry of Health guidelines for the care and use of animals (D. L. 4 March 2014 n. 26 and D. L. 27 January 1992 n. 116).

The sample size was based on the frequency of isolation of *Staphylococcus pseudintermedius* from the skin of a single body site in healthy dog skin (10), with a confidence level of 95%.

Based on this, five dogs per breeding kennel were sampled. Inclusion criteria were: clinically healthy adult female dogs of reproductive age, not treated with antimicrobials for at least 3 weeks prior to sampling.

Two samples were collected from each animal using sterile swabs (ESwab, 480 CE, Copan Italia Spa, Brescia): one from the perivulvar skin (swabbed for 3–5 s), and one from the rectum (swab gently introduced for about 2 cm and rotated for 3–5 s), in both cases without any previous disinfection. The swabs were rapidly placed in a vial filled with 1 mL of Liquid Amies Medium and sent refrigerated to the Istituto Zooprofilattico Sperimentale delle Venezie for processing within 48 h.

### 2.2 Bacterial culture

Isolation and identification of bacteria were completed in accordance with standard lab culture techniques. Antimicrobial susceptibility testing was carried out by broth micro-dilution to determine minimum inhibitory concentration (MIC) (15). Briefly, 10 μL aliquots of liquid carrier media of vulvar and rectal swabs were cultured as follows: i. streaked into a nutrient medium (5% sheep blood agar plates, AS, Biolife, Milan, Italy) and into a selective medium for Enterobacterales*”* (MacConkey Agar, McC, Biolife, Milan, Italy); ii. 100 μL aliquots were inoculated into selective broths: 6.5% NaCl Muller Hinton Broth (6.5% MHB, Biolife, Milan, Italy), to promote the growth of *Staphylococci* and Heart Infusion Broth (BHI, Biolifie, Milan, Italy) supplemented with 1 mg/L cefotaxime (CTX, Merck Life Science Darmstadt, Germania) to select the growth of cefotaxime-resistant *Enterobacteria*, suspected extended spectrum beta-lactamase (ESBL) producers.

Solid and liquid media were incubated at 37 °C for 18–24 h under aerobic conditions.

The solid media were then examined for coagulase-positive staphylococci and *E. coli* colonies. Ten μL aliquots from selective broths were streaked on selective media for methicillin-resistant staphylococci (MRS; CHROMAgar II MRSA, Becton Dickinson New Jersey, United States) and for ESBL-producing *Enterobacteria* (McConkey agar supplemented with 1 mg/L CTX) and incubated at 37 °C for 24–48 h under aerobic conditions. Colonies ranging from white to mauve on MRS-selective medium were isolated, identified and screened for the presence of the *mec*A gene using molecular biology methods (16).

A disk diffusion assay was performed on *E. coli* colonies grown on McC-CTX medium to phenotypically confirm ESBL-producing *E. coli* strains, following European Committee on Antimicrobial Susceptibility Testing guidelines (17). The content of the disks was the following: cefotaxime 30 μg, ceftazidime 30 μg, cefotaxime - clavulanic acid 30/10 μg, ceftazidime - clavulanic acid 30/10 μg, meropenem 10 μg, cefoxitin 30 μg, cefepime 30 μg.

Bacterial identification was performed by mass spectrometry (MALDI-TOF MS: Microflex LT instrument -MALDI Biotyper, Bruker Daltonics) equipped with FlexControl software (version 3.3, Bruker Daltonics).

The resistance profiles were determined by broth microdilution testing using specific antimicrobial panels for Gram-positive and Gram-negative bacteria (Thermo Scientific SensititreTM ITISVE6 and ITISVE7, Thermo Fisher Diagnostics, Segrate (MI) Italy) and Minimum Inhibitory Concentrations (MICs) were measured for:

- *Staphylococci*: Penicillins/Beta-lactam/Beta-lactamase inhibitor combinations (penicillin, ampicillin, amoxicillin-clavulanic acid, oxacillin), Cephems (first generation cephalosporin: cephalexin, cephazolin; third generation cephalosporin: cefpodoxime), Tetracyclines (tetracycline, doxycycline), Aminoglycosides (clindamycin, gentamycin, erythromycin, kanamycin), Phenicols (florphenicol), Fluoroquinolones (prototype: enrofloxacin), Folate pathway inhibitors (trimethoprim sulfamethoxazole).
- *E. coli*: Beta lactam/Beta lactamase inhibitor combinations (ampicillin, amoxicillin-clavulanic acid), Cephems (first generation cephalosporin: cephalexin, cephazolin; third generation cephalosporin: cefpodoxime, cefovecin), Tetracyclines (tetracycline, doxycycline), Aminoglycosides (gentamycin, erythromycin, kanamycin, amikacin), Fluoroquinolones (prototype: enrofloxacin), Folate pathway inhibitors (trimethoprim sulfamethoxazole).

The breakpoints used to define bacteria as resistant, intermediate or susceptible to each antimicrobial agent are shown in Table 1 and follow the Clinical and Laboratory Standards Institute (18, 19) and the epidemiological cutoff values (ECOFFs) of the European Committee on Antimicrobial Susceptibility Testing (20).

**Table 1:** Breakpoints used to define *E. coli* and *S. pseudintermedius* as resistant, intermediate or susceptible to each antimicrobial agent.

Antimicrobial	Microrganism	Lesion site	Species	S (mg/L)	SDD (mg/L)	I (mg/l)	R (mg/L)	Ref.	ECOFF (mg/L)****
Ampicillin	*E. coli*	ur	dog	≤ 8	-	-	-	*	8 (4–16)
Ampicillin	Enterobacterales	SST	dog	≤ 0.25	-	0.5	≥ 1	*	
Amoxicillin/clavulanic acid	*E. coli*	ur	dog	≤ 8/4	-	-	-	*	(8) (2–64)
Amoxicillin/clavulanic acid	Enterobacterales	SST	dog	≤ 0.25/0.12	-	0.5/0.25	≥ 1/0.5	*	
Cephalexin	*E. coli*	ur	dog	≤ 16	-	-	≥ 32	*	(32) (4–32)
Cephalexin	Enterobacterales	SST	dog	≤ 2	-	4	≥ 8	*	
Cephazolin	Enterobacterales	SST	dog	≤ 2	-	4	≥ 8	*	
Cefpodoxime	*E. coli*	ur, wrd, absc	dog	≤ 2	-	4	≥ 8	*	
Cefovecin	*E. coli*	ur	dog	≤ 2	-	4	≥ 8	*	
Tetracycline	Enterobacterales	n/a	human	≤ 4	-	8	≥ 16	*	
Doxycycline	Enterobacterales		dog	≤ 0.12	-	0.25	≥ 0.5	*	
Gentamycin	Enterobacterales	n/a	dog	≤ 2	-	4	≥ 8	*	
Kanamycin	Enterobacterales		human	≤ 16	-	32	≥ 64	*	
Amikacin	Enterobacterales	n/a	dog	≤ 4	-	8	≥ 16	*	
Enrofloxacin	Enterobacterales	SST, ur, resp	dog	≤ 0.06	0.12-	-	≥ 0.5	*	
Pradofloxacin	*E. coli*	SST, ur	dog	≤ 0.25	-	0.5–1	≥ 2	*	
Trimethoprim/sulfamethoxazole	Enterobacterales	n/a	human	≤ 2/38	-	-	≥ 4/76	*	
Penicillin	*Staphylococcus* spp.	n/a	human	≤ 0.12	-	-	≥ 0.25	*	
Ampicillin	*S. pseudintermedius*	SST	dog	≤ 0.25	-	-	≥ 0.5	*	
Amoxicillin/clavulanic acid	*Staphylococcus* spp.	SST, ur	dog	≤ 0.25/0.12	-	0.5/0.25	≥ 1/0.5	*	
Oxacillin	*S. pseudintermedius*	n/a	human	0.25	-	-	≥ 0.5	*	
Cephalexin	*S. pseudintermedius*	SST	dog	≤ 2	-	-	≥ 4	*	
Cephazolin	*S. pseudintermedius*	resp., SST, ur	dog	≤ 2	-	4	≥ 4	*	
Cefpodoxime	*S. pseudintermedius*	wds/ absc	dog	≤ 2	-	4	≥ 8	*	
Cefovecin	*S. pseudintermedius*	SST	dog	≤ 0.5		1	≥ 2	*	
Tetracycline	All staphylococci	SST	dog	≤ 0.25	-	0.5	≥ 1	*	
Doxycycline	*Staphylococcus* spp.	SST	dog	≤ 0.12	-	0.25	≥ 0.5	*	
Clindamycin	*Staphylococcus* spp.	SST	dog	≤ 0.5	-	1–2	≥ 4	*	
Gentamycin	All staphylococci	n/a	human	≤ 4	-	8	≥ 16	*	
Erythromycin	All staphylococci	n/a	human	≤ 0.5	-	1–4	≥ 8	*	
Florfenicol	*Staphylococcus intermedius*	skin	dog	≤ 4			≥ 8	***	
Kanamycin	All staphylococci	n/a	human	≤ 8	-	-	>8	**	
Amikacin	*Staphylococcus* spp.	n/a	dog	≤ 4	-	8	≥ 16	*	
Enrofloxacin	*Staphylococcus* spp.	resp., SST, ur	dog	≤ 0.06	0.12–0.25	-	≥ 0.5	*	
Pradofloxacin	*S. pseudintermedius*	skin, ur	dog	≤ 0.25	-	0.5–1	≥ 2	*	
Trimethoprim/sulfamethoxazole	*Staphylococcus* spp.	n/a	human	≤ 2/38	-	-	≥ 4/76	*	

resp., respiratory; SC, subcutaneously; SST, skin and soft tissue; ur, (lower) urinary tract; wds/absc, wounds and/or abscesses; n/a, not applicable; S, susceptible; SDD, susceptible-dose dependent; I, intermediate; R, resistant; ECOFF, epidemiological cutoff values. * (18); ** (46); *** (47); **** (20).

MDR was defined as acquired non-susceptibility to at least one agent in three or more antimicrobial categories (11).

### 2.3 Questionnaire

A structured questionnaire was administered directly to the dog breeders (the questionnaire is provided as Supplementary material), concurrently with the sample collection. The organization of the questions and answers was done by following the general template and indications of some other questionnaire-based studies already published for cat breeders (21). The dog breeders were allowed to respond in privacy and return the questionnaire before leaving the facility or send it digitally at a later date. The questionnaire consisted of multiple-choice questions related to the main characteristics of the breeding facility and to antimicrobial use, particularly around mating/artificial insemination/parturition. Questions were formulated to require categorical or numerical answers.

Breeding facility data (3 questions) covered the number of adult breeding animals, litters per year, and breeds maintained.

Questions on antimicrobial stewardship assessed factors influencing the initiation and termination of antimicrobial treatments, including the agents used, treatment duration, and decision-making authority (e.g., breeder, veterinarian).

The section on antimicrobial use investigated the frequency of use in different circumstances, with response options ranging from “always” to “never” (2 questions). Two more questions were included to assess antimicrobial administration at artificial insemination, mating or parturition, asking the active principles used, the route of administration, and treatment duration.

The questionnaire was developed with the aim to assess whether antimicrobials were used according to the Italian guidelines for antimicrobial use in small animals (22). Responses were recorded electronically and anonymised.

### 2.4 Analysis of data

The percentage of resistance of the isolated bacterial strains to the tested antimicrobial agents was calculated, as well as the prevalence of ESBL *E. coli*, *mec*A-positive *S. pseudintermedius* and of MDR strains.

Prior to the questionnaire administration to breeders, the answers to the categorical questions of the questionnaire were scored on a 1–5 scale: 1 indicated no antimicrobial use, and 5 indicated very frequent or uncontrolled use.

The breeding kennels were then categorized according to the antimicrobial-use score: M (minimal, ≤ 14): only in case of diagnosed pathological conditions, under veterinary supervision; S (seldom, 14–18): occasional use in suspected pathological conditions, usually under veterinary advice; and R (regular, ≥ 19): repeated use at mating/AI/parturition, often without veterinary consultation.

Statistical analysis was performed using Prism for Mac, Version 10.6.0 (GraphPad Software Inc., La Jolla, CA, United States).

The association between resistance percentages (summing up resistant and intermediate strains: R + I vs. S) of *S. pseudintermedius* and *E. coli* toward each antimicrobial and the breeding kennel category was assessed using Fisher’s exact test because of small expected counts. For each bacterial species, we considered the set of antibiotic-specific Fisher tests (resistant+intermediate *vs* susceptible by kennel antimicrobial-use score) as one family and controlled the false discovery rate using the Benjamini-Hochberg procedure at Q = 0.05.

The same test was applied to ESBL *E. coli*, methicillin-resistant *S. pseudintermedius* (MRSP) and MDR strain prevalence.

Significance was set at *p* < 0.05.

## 3 Results

The mean age (±standard deviation) of the bitches was 4.6 ± 2.3 years.

A total of 148 samples (74 perivulvar and 74 rectal) were collected. From these, 116 *E. coli* and 36 *S. pseudintermedius* strains were isolated and susceptibility testing was performed on 71 (uniformly distributed among kennels) and 34 strains, respectively.

**Figure 1:**
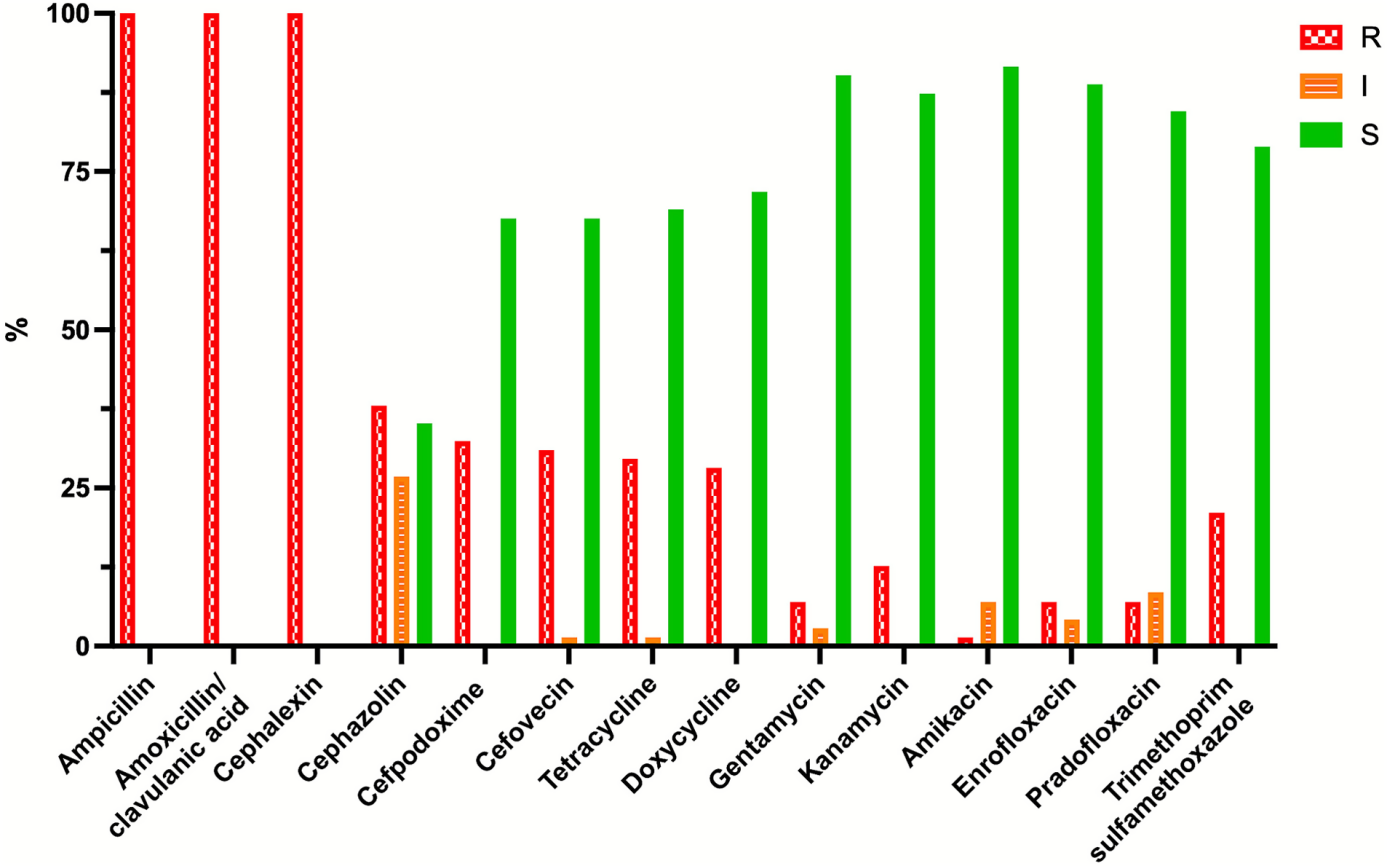
Percentage of resistant (R, red bars), intermediate (I, orange bars), and susceptible (S, green bars) *Escherichia coli* strains (*N* = 71) isolated from perivulvar and rectal area of the breeding bitches enrolled in the study. Breakpoints for ampicillin, amoxicillin-clavulanic acid, and cefalexin: dog lower urinary tract (18).

**Figure 2:**
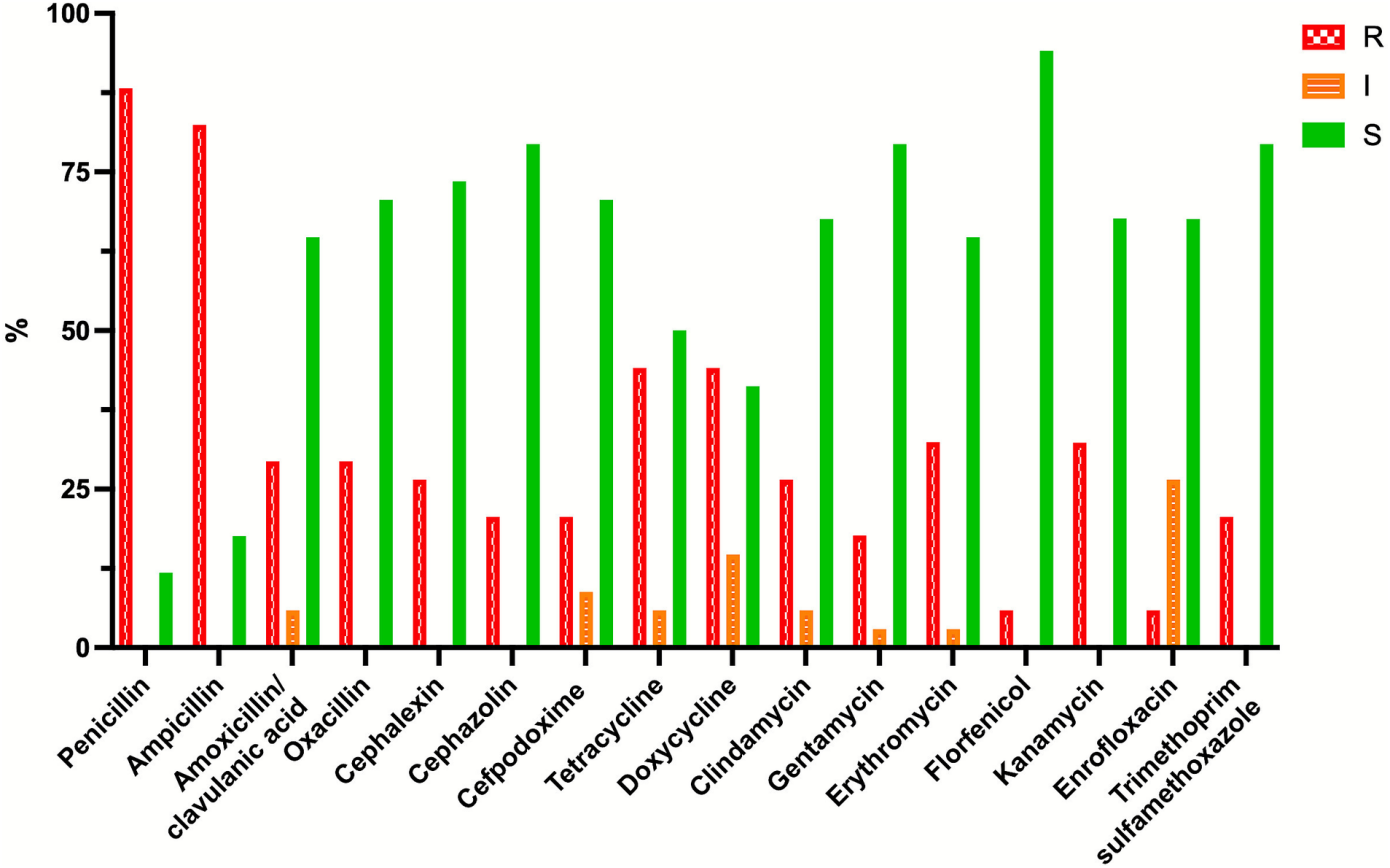
Percentage of resistant (R, red bars), intermediate (I, orange bars), and susceptible (S, green bars) *Staphylococcus pseudintermedius* strains (*N* = 34) isolated from perivulvar and rectal areas of the breeding bitches enrolled in the study.

The resistance profiles of the two bacteria are shown in Figures 1, 2. When using dog skin/soft tissue breakpoints, all *E. coli* isolates were resistant to ampicillin, amoxicillin/clavulanic acid and cephalexin, while less than 16% were resistant to the tested fluoroquinolones. With the dog urinary tract breakpoints, 52.1, 76.1 and 67.6% of the *E. coli* isolates were susceptible to ampicillin, amoxicillin/clavulanic acid and cephalexin, respectively. Specifically, when lower than canine urinary tract breakpoints, the MICs of *E. coli* for amoxicillin-clavulanic acid were the following: ≤1/0.5 2.8% of isolates; ≤2/1 8.5% of isolates; ≤4/2 36.6% of isolates; for ampicillin: ≤1 1.4%; ≤2 16.9%; ≤4 25.4%; for cefalexin ≤8 60.6% of isolates. All these isolates are within the ECOFFs breakpoints defined for wildtype *E. coli*.

Over 82% *S. pseudintermedius* strains were resistant to penicillin and ampicillin, while resistance to amoxicillin/clavulanic acid and the tested cephalosporins was lower (between 20 and 30%).

The prevalence of phenotypically ESBL *E. coli* was 7.76% (*N* = 9/116), while *mec*A-positive *S. pseudintermedius* accounted for 17.65% (*N* = 6/34). Notably, 4 out of the 6 *mec*A-positive MRSP isolates came from a single breeding kennel.

MDR *E. coli* strains represented 22.53% of the tested isolates (*N* = 16/71) and MDR *S. pseudintermedius* strains represented 41.18% of the tested isolates (*N* = 14/34).

Three breeding kennels fell into the ‘R’ category (excessive antimicrobial use), nine into the ‘S’ category and two in the ‘M’ one (minimal use). One breeder did not respond to this section of the questionnaire.

Significantly higher percentages of *E. coli* isolates resistant to doxycycline (*p* < 0.0001, q = 0.0011) and tetracycline (*p* = 0.0011, q = 0.0058) were found in both M and R breeding kennels. Lower resistance percentages to trimethoprim-sulfamethoxazole were found in the S kennels (*p* = 0.0049, q = 0.0129). Higher resistance to cephazolin was found in M kennels (100%, *p* = 0.0020, q = 0.007). No significant association was found between the kennel category and the percentage of resistance toward any of the antimicrobials tested for *S. pseudintermedius*.

Similarly, the prevalence of ESBL *E. coli*, *mec*A-positive *S. pseudintermedius* and MDR *S. pseudintermedius* was not associated with the breeding kennel category. MDR *E. coli* was significantly more frequent in R kennels (*p* = 0.0160). ESBL *E. coli* were found across all kennel categories. Although no MRSP was isolated in ‘M’ breeding kennels, MRSP strains were detected in varying proportions in both ‘R’ and ‘S’ facilities.

**Figure 3:**
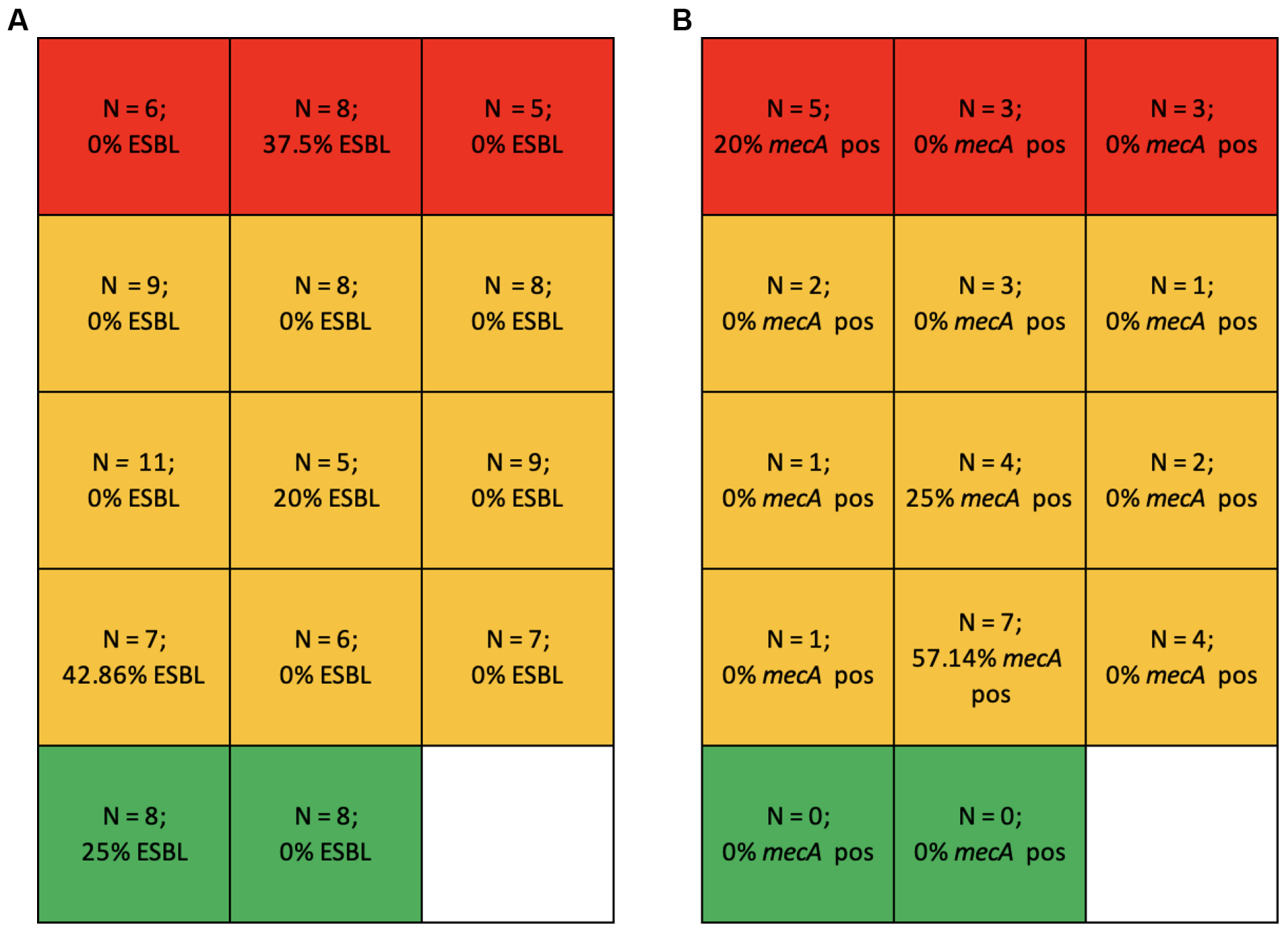
**(A)** Number of isolated *E. coli* strains (*N*) and percentage of ESBL *E. coli* in the different kennel categories: R (excessive use), red; S (occasional use), orange; M (minimum use), green. **(B)** Number of isolated *S. pseudintermedius* strains (*N*) and percentage of *S. pseudintermedius mecA* positive strains in the different kennel categories: R (excessive use), red; S (occasional use), orange; M (minimum use), green.

Figure 3 reports the prevalence of ESBL *E. coli* (Figure 3A) and of *mec*A-positive MRSP (Figure 3B) by breeding kennel category.

## 4 Discussion

The overuse of antimicrobials drives the development of bacterial resistance, eventually resulting in limited therapeutic options. Surveillance of AMR is therefore essential, with epidemiological investigations forming the foundation of rational control strategies. Although the risk of AMR is often primarily associated with food-producing animals (23), companion animals can also serve as reservoirs and sources of transmission of resistance genes and/or resistant bacteria to humans (7). In collective housing systems such as breeding facilities, high density, inadequate hygiene, structural deficiencies, and suboptimal management practices can promote antimicrobial use, thereby selecting for resistant bacteria (24). Moreover, in the dog breeding sector, antimicrobials are sometimes misused for prophylactic purposes or administered to enhance fertility at mating or artificial insemination in case of suspected infectious infertility (25), as well as to reduce neonatal mortality around parturition (24). Consequently, breeding facilities are frequently characterized by a higher prevalence of antimicrobial-resistant bacteria (15, 26).

Our findings from Northern Italy are concerning, as both indicator species exhibited high levels of acquired resistance to multiple antimicrobials, reflecting substantial antimicrobial exposure in the studied population. Surprisingly, over 20% *E. coli* and 38% *S. pseudintermedius* isolates were MDR, underscoring the intense selective pressure exerted by antimicrobial use.

Comparable investigations in healthy dogs are limited and are generally not directly comparable with our findings, due to differences in the breakpoints used to define resistance (27, 28), variability in geographical regions (29) and heterogeneity of dog populations, which often include privately owned pets or a mixture of healthy and clinical cases spanning broad age ranges (27). Breakpoints for defining *E.coli* susceptibility to beta-lactam antimicrobials vary substantially depending on the bacterial source: breakpoints for canine skin and soft tissue are considerably lower than those for the canine urinary tract.

Given that we sampled healthy dogs with no recent antimicrobial treatment, lower resistance rates were anticipated. However, when applying canine skin and soft tissue breakpoints, all *E. coli* isolates were classified as resistant to ampicillin, amoxicillin-clavulanic acid, and cephalexin. When using the higher breakpoints established for the canine lower urinary tract, over 50% of *E. coli* isolates were reclassified as susceptible to the three antimicrobials and fell within the cut-off values defining wildtype microorganism (20). Resistance to amoxicillin-clavulanic acid (23.9%) was also lower than that reported by Siugzdaite et al. (28) in Lithuania (39.6%), who applied even higher breakpoints. These results may suggest that beta-lactam overuse is not widespread in our studied population.

Administration of amoxicillin has been shown to select for resistance in commensal intestinal canine *E. coli* (30, 31) whereas treatment with amoxicillin-clavulanic acid has been associated not only with increased resistance to this drug, but also to chloramphenicol, nalidixic acid, tetracycline, trimethoprim, as well as with a higher percentage of MDR strains (32).

In our study, approximately 16% of *E. coli* isolates were resistant to enrofloxacin, exceeding the 9.2% reported in healthy dogs in Chile (29). This difference may suggest a greater use of fluoroquinolones in our region, despite the geographic disparity.

The high resistance of *S. pseudintermedius* to penicillin and ampicillin reflects beta-lactam use in the investigated breeding kennels, although overuse appears unlikely, given that resistance to amoxicillin-clavulanic acid remained below 30% and the prevalence of *mec*A positive MRSP was relatively low. Notably, 4 of 6 *mec*A-positive MRSP isolates originated from a single kennel, suggesting the influence of localized factors. Such clustering of MRSP isolates in specific breeding facilities has been previously reported in Italy (15).

Reported MRSP prevalence in healthy dogs varies widely (0–60%), but comparisons are limited by differences in geography (33–35) and sample-size (36, 37). Data from breeding kennels indicate prevalence as high as 55% in bitches with reproductive disorders in a Lithuanian study (38), whereas healthy breeding bitches in the southwestern United States showed a prevalence of only 1.9% MRSP (39).

Two encouraging findings were the low prevalence of phenotypic ESBL *E. coli* (7.76%) and the relatively low rates of fluoroquinolone resistance, suggesting limited use of this antimicrobial class. Fluoroquinolone-resistance arises primarily from fluoroquinolone administration, but it can also result from exposure to cefalexin or cefovecin treatment, which may induce cross-resistance to fluoroquinolones (32). In dogs treated with enofloxacin, *E. coli* isolates exhibited resistance not only to fluoroquinolones, but also to beta-lactams, aminoglycosides, tetracyclines and phenicols, with a reported 20% prevalence of ESBL strains (40). Similarly, higher colonization by MDR *E. coli* strains following oral enrofloxacin administration has been documented in previous studies (41).

Prudent antimicrobial use requires administration only when clinically indicated. However, in canine reproduction, prophylactic antimicrobial use remains common, particularly for infertility management or to prevent neonatal mortality. Veterinarians who are not specialized in reproductive medicine may face pressure from breeders requesting antimicrobial administration, and often comply with it to avoid being held responsible for unsuccessful breeding outcomes (42). For suspected infectious infertility, antimicrobials are typically administered around mating. Even when vaginal cultures are performed, interpretation is challenging as isolation of bacteria from the canine vagina does not necessarily indicate reproductive tract infection or a causative factor for infertility (25, 43).

A secondary objective of this study was to assess antimicrobial use practices in breeding dog management. Breeder responses, however, were partially inconsistent, particularly when compared with AMR prevalence results. For instance, ‘virtuous’ kennels reporting minimal antimicrobials use paradoxically exhibited higher *E. coli* resistance to some antimicrobial agents. Conversely, kennels reporting high antimicrobial use showed a correspondingly high prevalence of MDR *E. coli* strains. The low number of *S. pseudintermedius* isolates limited the statistical power of the analysis, and no associations reached statistically significance.

A possible interpretation of these controversial results may relate to several limitations in the questionnaire design and administration, the questions formulation and the answers scoring. A key weakness was the lack of anonymity during completion, which could have introduced social desirability bias; this was necessary, however, to link breeders’ responses with the corresponding bacteriological results. The scoring system of the responses could be refined to better capture the intensity of antimicrobials use. Inclusion of a larger number of breeding facilities would have increased both the statistical power and the representativeness of the results. Future studies should involve larger samples, a more structured and anonymized questionnaire protocol, and, where possible, incorporation of veterinary records or prescription data. Additionally, alternative analytical approaches to detect antimicrobial administration in dog breeding facilities could help obtain more robust and consistent results.

This cross-sectional study provides an assessment of AMR in commensal *E. coli* and *S. pseudintermedius*, used as indicator species (44, 45). However, resistance patterns of commensal populations are dynamic and may decline following the withdrawal of antimicrobial pressure, as demonstrated for enrofloxacin-resistant fecal coliforms (41) and for *mec*A-positive MRsp., whose persistence reflects ongoing antimicrobial exposure (6).

Overall, our findings indicate a relatively high antimicrobial pressure combined with limited adherence to established principles of prudent antimicrobial use among breeders. Veterinarians, particularly those involved in canine reproduction, should be trained not only in appropriate antimicrobial use but also in managing breeders’ expectations and avoiding the prescription of antimicrobials as substitutes for addressing inadequate management practices or suboptimal facility conditions. Furthermore, consensus guidelines for antimicrobial use in the management of fertility in small animals breeding facilities should be developed by specialists in small animal reproduction.

## 5 Conclusion

The detection of antimicrobial resistance in both indicator bacterial species suggests substantial antimicrobial use in the investigated breeding facilities. Moreover, the concentration of *mec*A-positive MRSP within individual facilities underlines a heterogeneous potentially inappropriate antimicrobial use. The inconsistencies observed in breeders’ questionnaire responses further indicate that more robust monitoring approaches are required to accurately assess antimicrobial use in this setting.

Although limited by the small number of breeding facilities and the partial inconsistency of the breeders’ responses, this study contributes valuable baseline data to a field that remains poorly investigated and underscores the need for targeted interventions. Overall, our findings reinforce the necessity of strengthening antimicrobial stewardship in breeding facilities and highlight the importance of increasing awareness among both breeders and veterinarians.

## Data Availability

The raw data supporting the conclusions of this article will be made available by the authors, without undue reservation.
