# Supplementary material for: Antimicrobial use in breeding kennels and antimicrobial resistance profile of Escherichia coli and Staphylococcus pseudintermedius isolated from healthy breeding bitches in Northern Italy
Source: Front Vet Sci. 2026 Jan 12;12:1703350. doi: 10.3389/fvets.2025.1703350 (PMC12832429; doi:10.3389/fvets.2025.1703350)
Supplement: Supplementary file 1 [file Data_Sheet_1.docx]

Supplementary Material

# Supplementary Table 1

| **Questionnaire for dog breeders** | |
| --- | --- |
| **Specific questions for dog breeders**   1. How much do you agree with the following statements about beginning antibiotic treatments? (100%, 75%, 50%, 25%, 0%) 2. I treat with antibiotics when prescribed by my vet 3. I treat with antibiotics when advised by a fellow breeder 4. I treat with antibiotics if I have the impression that the dog is unwell 5. I treat with antibiotics if I notice that the dog has similar symptoms to previous episodes when antibiotics were prescribed 6. I treat with antibiotics if I notice that the dog has similar symptoms to other dogs that have been prescribed antibiotics 7. When I treat a dog with antibiotics, I also treat the other dogs that share its living spaces 8. I treat before artificial insemination 9. I treat before natural mating 10. I treat before the expected date of parturition 11. How much do you agree with the following statements about interrupting antibiotic treatments? (100%, 75%, 50%, 25%, 0%) 12. I stop giving antibiotics when my vet or the prescription indicates it 13. I stop giving antibiotics earlier than recommended by my vet or than indicated on the prescription 14. I stop giving antibiotics when advised by a fellow breeder 15. I stop giving antibiotics when the dog’s treated symptoms seem to have improved 16. If you use antibiotics before artificial insemination or natural mating, please indicate how you usually administer them (you may select more than one answer):     1. Drugs used:        1. Amoxicillin + clavulanic acid (e.g., Synulox, Konclav, Kesium, Clavaseptin, Cylanic, etc.)        2. Cephalosporins (e.g., ICF Vet, Cefabactin, Cefa-Cure, Convenia, etc.)        3. Metronidazole (e.g., Metrobactin, etc.)        4. Enrofloxacin or other fluoroquinolones (e.g., Baytril, Marbocyl, Aristos, etc.)     2. Treatment duration:        1. 3–5 days        2. 1 week        3. 2 weeks        4. More than 2 weeks     3. Route of administration:        1. Oral        2. Subcutaneous        3. Intramuscular 17. If you treat before the expected whelping date, please indicate how you usually administer antibiotics (you may select more than one answer):     1. Drugs used:        1. Amoxicillin + clavulanic acid (e.g., Synulox, Konclav, Kesium, Clavaseptin, Cylanic, etc.)        2. Cephalosporins (e.g., ICF Vet, Cefabactin, Cefa-Cure, Convenia, etc.)        3. Metronidazole (e.g., Metrobactin, etc.)        4. Enrofloxacin or other fluoroquinolones (e.g., Baytril, Marbocyl, Aristos, etc.)     2. Treatment duration:        1. 3–5 days before the expected date        2. 1 week before the expected date        3. 2 weeks before the expected date        4. More than 2 weeks before the expected date     3. End of therapy:        1. On the day of parturition        2. 3-5 days after parturition        3. 1 week after parturition        4. 2 or more weeks after parturition     4. Route of administration:        1. Oral        2. Subcutaneous        3. Intramuscular | **Socio-demographic information**   1. Age: 2. 20-30 years 3. 30-40 years 4. 40-50 years 5. 50-60 years 6. > 60 years 7. Gender: 8. Female 9. Male    1. Which dogs do you currently breed? (please specify the breed[s])    2. How many breeding dogs are there in your kennel?    3. How many litters per year?    4. In which province is the kennel where you work located? 10. Padua 11. Venice 12. Verona 13. Vicenza 14. Rovigo 15. Turin 16. Alessandria     1. Education level: 17. Primary school 18. Middle school (lower secondary level) 19. Secondary school (upper secondary level) 20. Vocational qualification 21. University degree     1. Employment status:        1. Student        2. Homemaker        3. Unemployed        4. Employed        5. Retired     2. How long have you been working in dog-breeding facilities?        1. 1 year        2. 2–3 years        3. 5 years        4. >10 years |
